# Left Ventricular Ejection Fraction in Patients Undergoing Transcatheter Aortic Valve Implantation: Insights From the COMPARE-TAVI-1 Study

**DOI:** 10.1016/j.jacadv.2025.102547

**Published:** 2026-01-24

**Authors:** Preman Kumarathurai, Christian Juhl Terkelsen, Henrik Nissen, Jonas Agerlund Povlsen, Henrik Vase, Mathias Dyreborg Jørgensen, Kristian Laursen, Anders Lehman Dahl Pedersen, Troels Thim, Ashkan Eftekhari, Philip Freeman, Frederik Uttenthal, Ulrik Christiansen, Evald Høj Christiansen, Jordi Sanchez Dahl

**Affiliations:** a Department of Cardiology, Odense University Hospital, Odense, Denmark; b Department of Clinical Research, University of Southern Denmark, Denmark; c Department of Cardiology, Aarhus University Hospital, Aarhus, Denmark; d Department of Clinical Medicine, Aarhus University Hospital, Aarhus, Denmark; e Department of Cardiology, Aalborg University Hospital, Aalborg, Denmark; f Department of Cardiovascular Medicine, Mayo Clinic, Rochester, Minnesota, USA

**Keywords:** aortic stenosis, echocardiography, heart failure, left ventricular ejection fraction, transcatheter aortic valve implantation

## Abstract

**Background:**

Reduced LV ejection fraction (LVEF) in aortic stenosis may result from afterload mismatch or reduced contractility. Limited data exist on post-transcatheter aortic valve implantation (TAVI) LVEF changes and factors linked to persistent low LVEF.

**Objectives:**

The aim of this substudy was to identify variables associated with persistent low LVEF after TAVI.

**Methods:**

The COMPARE-TAVI 1 trial is an all-comer study in which patients scheduled for transfemoral TAVI were randomized 1:1 to receive either the Sapien 3/Sapien 3 Ultra or Myval/Myval Octacor transcatheter heart valve. Patients were categorized into 2 groups based on their baseline LVEF: low LVEF (≤40%) or normal LVEF (>40%). Serial LVEF were analyzed alongside 12-month mortality.

**Results:**

Of 1,029 patients, men comprised 60%. The mean LVEF was 55% ± 14%. Among patients with low baseline LVEF (n = 151, 15%), the mean LVEF improved from 31% ± 6% to 45% ± 15% at 1 month, and 52% ± 14% at 12 months (*P* < 0.001). However, 20% of these patients had persistent LVEF ≤40% at 1 year, while 5% of patients with normal baseline LVEF declined to ≤40%. Chronic heart failure, pacemaker pre-TAVI, baseline LVEF, left ventricular end-diastolic diameter, aortic mean gradient, and stroke volume index were associated with persistent low LVEF at 1 year. No significant differences in 1-year mortality were observed between groups.

**Conclusions:**

TAVI improves LVEF in the majority of patients with low baseline LVEF. However, a subset of patients experiences persistent left ventricular impairment, with chronic heart failure, pacemaker, and baseline LVEF as key predictors.

Left ventricular (LV) systolic dysfunction is recognized to be an adverse result of the pressure overload that occurs in severe aortic stenosis (AS). The increased LV wall stress in AS leads to LV hypertrophy, concentric remodeling, compromised coronary flow reserve^1^ and eventually reduced LV systolic function that is uncommonly asymptomatic.^2^ LV ejection fraction (LVEF) is the most routinely used parameter when assessing LV systolic function; in AS, it is, however, well known that LVEF may remain normal despite reduced myocardial contractility by use of preload reserve^3^ or changes in LV geometry.^4^ In contrast, a decreased LVEF may occur in the setting of preserved contractility due to an afterload mismatch,^3^^,^^5^, ^6^, ^7^ but could also represent a failing LV.^6^ According to both American College of Cardiology and European Society of Cardiology valvular heart disease guidelines, LVEF<50%, is considered a class I indication for aortic valve replacement (AVR) in severe AS, even in the patient without symptoms.^8^^,^^9^

However, while patients with reduced LVEF due to afterload mismatch may recover fully after AVR, patients with LV failure prior to AVR may have persisting heart failure symptoms and systolic heart failure after AVR.^10^ Although differentiating these 2 entities seems imperative as patients with LVEF ≤40% irrespectively of AS largely benefit from guideline-directed medical therapy for heart failure with reduced ejection fraction,^11^ there is a paucity in data describing which patients should be referred for heart failure clinics to commence heart failure medical therapy.

Thus, the aim of this study was to describe changes in LVEF after transcatheter aortic valve implantation (TAVI) and to find variables able to identify patients at risk for having systolic heart failure 12 months after TAVI.

## Methods

The COMPARE-TAVI 1 trial is a multicenter, prospective, randomized controlled trial, in which all-comer patients were randomized 1:1 to receive transfemoral TAVI with either Sapien 3/Sapien 3 Ultra Transcatheter Heart Valve series (Edwards Lifesciences) or the Myval/Myval Octacor THV series (Meril Life Sciences Pvt Ltd).^12^

The study has received ethical approval national from the Central Denmark Region. Written and verbal informed consent was obtained from all screened participants at the time of enrollment. The study was conducted in accordance with the Declaration of Helsinki, and the main results have been published.^12^

Patient characteristics were collected at baseline, while clinical and echocardiographic follow-up was performed at baseline, before discharge, after 30 days, and 1 year.

Clinical characteristics, including comorbidities and device information, were site-reported by the participating centers and obtained from patient medical records. Data-quality checks were performed to ensure completeness and internal consistency, but information was not independently adjudicated.

Ischemic heart disease (IHD) was defined as a composite variable including a history of previous acute myocardial infarction, percutaneous coronary intervention, or coronary artery bypass grafting. Patients without a history of these conditions who were managed conservatively for stable IHD were not classified as having IHD.

Information on pacemaker device type at baseline was obtained from clinical records. Devices were categorized as pacemaker, implantable cardioverter-defibrillator (ICD), cardiac resynchronization therapy (CRT), or CRT-ICD. For analysis, we grouped pacemaker and ICD devices together as the right ventricular (RV) pacing device group, reflecting single- or dual-chamber pacemaker systems primarily associated with RV pacing. Similarly, CRT and CRT-ICD devices were combined into a CRT device group, representing biventricular pacing systems. This grouping was used for all subsequent analyses.

To characterize postprocedural changes in LV systolic function, patients were categorized into 4 groups according to the trajectory of LVEF from baseline to 12-month follow-up: persistent low LVEF: patients with LVEF <40% both at baseline and at 12 months; recovered LVEF: patients with baseline LVEF <40% who improved to LVEF ≥40% at 12 months; persistent normal LVEF: patients with LVEF ≥40% at both baseline and 12 months; deteriorated LVEF: patients with baseline LVEF ≥40% who experienced a decline in LVEF to <40% at 12 months.

### Echocardiography

A comprehensive transthoracic echocardiography was performed according to a prespecified core laboratory echocardiographic protocol using Vivid 9 (GE Healthcare) or EPIQ7 (Philips Professional Healthcare) machines, digitally archived locally, deidentified and transferred in raw format to a core laboratory. The echocardiograms were performed at baseline before the TAVI procedure, at discharge, and at 1 and 12 months. Echocardiograms were analyzed by 5 readers that included highly experienced research fellows and board-certified cardiologists with level III certification in echocardiography, and were subsequently approved by the core lab director. Echocardiograms were analyzed using Viewpoint 6 (GE Healthcare) and Intellispace Cardiovascular (Philips Healthcare Best) software.

Doppler values were averaged over 3 cardiac cycles for patients with sinus rhythm and 5 cycles in those with atrial fibrillation, ensuring horizontal sweep of 100 cm/s and a frame rate of minimum 60/s. The aortic valve area was derived using the continuity equation, with peak and mean flow velocities determined from continuous-wave Doppler, ensuring alignment of the cursor as parallel as possible to the blood flow. Peak and mean transvalvular gradients were estimated using the modified Bernoulli equation.^13^ LVEF was calculated by the Simpson biplane method from the apical 4-chamber and 2-chamber views. Patients were stratified according to LVEF into those with LVEF ≤40% and LVEF >40%. LV stroke volume index (SVi) was calculated using pulsed-wave Doppler as the product of the LV outflow area and LV outflow tract time velocity integral indexed to body surface area. A low-flow condition was defined as SVi < 35 mL/m^2^. LV mass index was estimated using the Devereux formula.^14^ In men, LV mass index >115 g/m^2^ and in women >95 g/m^2^ were considered indicative of LV hypertrophy. Relative wall thickness was calculated for assessment of LV geometry using the formula 2× LV posterior wall thickness/LV internal diameter in diastole. Relative wall thickness was considered increased when >0.42. End-systolic wall stress was calculated using the following formula: (0.334 × [systolic blood pressure + aortic mean gradient] × LVESD)/(posterior wall diameter × [1 + posterior wall diameter/LV end-systolic diameter]). Patients were classified as normal geometry, concentric geometry, concentric LV hypertrophy, and eccentric LV hypertrophy based on LV mass index and relative wall thickness.^15^ Indexed left atrial volume and diastolic dysfunction grade were evaluated according to guidelines.^16^

### Statistics

Continuous variables are presented as mean ± SD or median (IQR), as appropriate. Categorical variables are expressed as counts and percentages. Group comparisons between patients with low LVEF ≤40% and normal LVEF >40% were performed using Student’s *t*-test or the Wilcoxon rank-sum test for continuous variables, depending on normality distribution assessed visually or by the Shapiro-Wilk test. Chi-square tests or Fishe exact tests were used for categorical variables.

Kaplan-Meier curves were constructed to compare 1-year all-cause mortality between low-LVEF and normal-LVEF groups. Survival differences were assessed using the log-rank test. Cox proportional hazards regression models were used to estimate HRs and 95% CIs for mortality. The proportional hazards assumption for LVEF group was evaluated using Schoenfeld residuals and was not violated (global test *P* = 0.18).

To identify predictors of persistent low LVEF (≤40%) at 1 year, univariate and multivariate logistic regression models were performed. Model 1 adjusted for baseline clinical factors (age, sex, weight, hypertension, diabetes), while model 2 was fully adjusted for additional variables: chronic heart failure (CHF), NYHA functional class, and atrial fibrillation. ORs and 95% CI were reported.

We evaluated several clinically plausible interaction terms, including baseline LVEF × CHF, baseline LVEF × NYHA functional class, mean aortic gradient × CHF, SVi × CHF. None of the interaction terms reached statistical significance; therefore, no interaction terms were retained in the final multivariable model. Multicollinearity was assessed using variance inflation factors (all <2), indicating no meaningful collinearity.

Missing data were low (<1% for baseline clinical variables). The proportion of missing LVEF measurements was also minimal when considering only patients who were alive at each follow-up. At baseline, 2 of 1,031 living patients (0.2%) had missing LVEF values and were excluded from subsequent analyses. At 12 months, 976 patients were alive and eligible for follow-up echocardiography (8 of whom died at 12 months after their study echocardiography). LVEF measurements at 12 months were available in 955 patients, corresponding to 21 missing values (2.2%). Given the minimal degree of missing data, complete-case analysis was used for all regression models, and no imputation was performed.

#### Sensitivity analysis

To assess the robustness of the findings, a sensitivity analysis was performed in which patients who died within the first 30 days following TAVI were excluded. The multivariable logistic regression model was then repeated using the same covariates as in the primary analysis.

All analyses were performed using Stata version 18 (StataCorp). A 2-tailed *P* value <0.05 was considered statistically significant.

## Results

Of the 1,031 patients included in the study, baseline echo images were available for core lab analysis in 1,029. The mean LVEF for the overall population was 55.1% ± 13.7%. In the low-LVEF group (n = 151, 15%), the mean LVEF was 30.9% ± 6.3%, and for the normal-LVEF group (n = 878, 85%), the mean LVEF was 59.3% ± 10.8%.

Patients with Low LVEF were more likely men; were more symptomatic with higher percentage of NYHA functional class III and IV; had lower systolic blood pressure; and with higher rates of CHF, left bundle branch block, and a history of pacemaker before TAVI. RV pacing device accounted for the majority of the device types in both groups. IHD accounted for 24.5% of the population with a nonsignificant higher proportion in the low-LVEF group compared to the normal-LVEF group (27.2% vs 24.1%, respectively; *P* = 0.43). The list of medications, including guideline-directed medical therapy, is provided, and notably, there was greater use of angiotensin receptor–neprilysin inhibitors, beta-blockers, sodium glucose co-transporter 2 inhibitor, and loop diuretics in the low-LVEF group (Table 1). Impaired longitudinal deformation with global longitudinal strain <15.0% was common occurring in 71% of the population. Still, compared to patients with LVEF ≥40%, those with LVEF <40% demonstrated lower longitudinal deformation, as measured by global longitudinal strain, and diastolic function, with shorter deceleration time and a higher early diastolic mitral inflow velocity ratio (Table 2). Patients with low LVEF had significantly larger LV dimensions and larger LV mass index, resulting in difference in LV geometry with eccentric remodeling pattern being the most common in patients with LVEF <40%. However, still patients with low LVEF had a lower SVi (31 ± 10 mL/m^2^ vs 40 ± 11 mL/m^2^, *P* < 0.001), suggestive of a lower cardiac output state.

**Table 1:** Baseline Characteristics

	Baseline LVEF ≤40% (n = 151)	Baseline LVEF >40% (n = 878)	*P* Value
Male, n (%)	103 (68%)	511 (58%)	**0.021**
Age, years, mean (SD)	80 (7)	81 (6)	0.16
Weight, kg, mean (SD)	77 (16)	80 (17)	0.15
Systolic BP (mm Hg), mean (SD)	127 (21)	141 (20)	**<0.001**
Heart rate, mean (SD)	78 (14)	71 (13)	**<0.001**
eGFR, mean (SD)	57 (19)	64 (19)	**<0.001**
EURO score 2, median (IQR)	5.6 (2.9, 9.2)	2.3 (1.5, 3.7)	**<0.001**
STS score, median (IQR)	3.0 (1.9, 4.7)	2.35 (1.5, 3.4)	**<0.001**
Valve morphology			
Valve in valve	14 (9.3%)	26 (3.0%)	**<0.001**
Bicuspid	16 (10.6%)	81 (9.2%)	
Tricuspid	121 (80.1%)	771 (87.8%)	
Diabetes mellitus, n (%)	37 (24.5%)	186 (21.2%)	0.36
CHF, n (%)	101 (66.9%)	108 (12.3%)	**<0.001**
NYHA functional class, n (%)			
I	5 (3.3%)	84 (9.6%)	**<0.001**
II	55 (36.7%)	474 (54.0%)	
III	81 (54.0%)	314 (35.8%)	
IV	9 (6.0%)	5 (0.6%)	
Atrial fibrillation, n (%)	65 (43.0%)	312 (35.5%)	0.08
Previous AMI, n (%)	18 (11.9%)	91 (10.4%)	0.57
Previous PCI, n (%)	32 (21.2%)	163 (18.6%)	0.45
Previous CABG, n (%)	17 (11.3%)	60 (6.8%)	0.056
Previous stroke, n (%)	19 (12.6%)	139 (15.8%)	0.31
Left bundle branch block, n (%)	25 (17.2%)	68 (7.9%)	**<0.001**
Pacemaker status before TAVI			
RV pacing device	19 (12.6%)	84 (9.6%)	**<0.001**
CRT device	4 (2.6%)	1 (0.1%)	
No pacemaker	128 (84.8%)	793 (90.3%)	
Hypertension, n (%)	104 (69.3%)	673 (76.7%)	0.05
Medication			
ACEi/ARB	88 (58.3%)	469 (53.4%)	0.27
ARNi	2 (1.3%)	0 (0.0%)	**<0.001**
Beta-blockers	80 (53.0%)	383 (43.6%)	**0.033**
MRA	16 (10.6%)	62 (7.1%)	0.13
SGLT2i	17 (11.3%)	27 (3.1%)	**<0.001**
Loop diuretics	118 (78.1%)	377 (42.9%)	**<0.001**
Digoxin	9 (6.0%)	41 (4.7%)	0.50
Hydralazine	1 (0.7%)	1 (0.1%)	0.16
CCB group II	45 (29.8%)	264 (30.1%)	0.95
Statin	83 (55.0%)	539 (61.4%)	0.14

ACEi = angiotensin-converting enzyme inhibitors; AMI = acute myocardial infarction; ARB = angiotensin receptor blockers; ARNi = angiotensin receptor–neprilysin inhibitors; BP = blood pressure; CABG = coronary artery bypass grafting; CCB = calcium channel blockers; CHF = chronic heart failure; CRT = cardiac resynchronization therapy; eGFR = estimated glomerular filtration rate; EUROscore 2 = The European System for Cardiac Operative Risk Evaluation 2; LVEF = LV ejection fraction; MRA = mineralocorticoid receptor antagonist; PCI = percutaneous coronary intervention; RV = right ventricular; SGLT2i = sodium glucose co-transporter 2 inhibitor; STS = Society of Thoracic Surgeons; TAVI = transcatheter aortic valve implantation.

Significant differences (*P* < 0.05) are highlighted in **bold**.

**Table 2:** Baseline Echocardiographic Characteristics

	Baseline LVEF ≤40% (n = 151)	Baseline LVEF >40% (n = 878)	*P* Value
Aortic peak velocity, cm/s, mean (SD)	379.8 (71.2)	430.0 (63.0)	**<0.001**
Aortic mean gradient (mm Hg), mean (SD)	38.1 (14.0)	49.5 (18.3)	**<0.001**
Aortic valve area (cm^2^), mean (SD)	0.72 (0.39)	0.73 (0.23)	0.71
LV outlet tract VTI (cm), mean (SD)	16.6 (5.1)	22.9 (5.7)	**<0.001**
Stroke volume index (mL/m^2^), mean (SD)	30.8 (9.7)	40.1 (11.3)	**<0.001**
LVEF, %, mean (SD)	30.9 (6.3)	59.3 (10.8)	**<0.001**
LV end-diastolic diameter (mm), mean (SD)	52.0 (7.8)	45.9 (7.0)	**<0.001**
LV end-systolic diameter (mm), mean (SD)	45.1 (8.6)	33.2 (8.6)	**<0.001**
LV mass index g/m^2^, mean (SD)	140.3 (31.6)	118.2 (29.5)	**<0.001**
End systolic wall stress, mean (SD)	161.5 (65.3)	131.5 (61.7)	**<0.001**
Relative wall thickness, %, mean (SD)	0.51 (0.16)	0.56 (0.18)	**<0.001**
GLS, %, mean (SD)	8.3 (2.7)	13.7 (3.1)	**<0.001**
LA volume index (mL/m^2^), mean (SD)	50.1 (18.3)	45.1 (17.7)	**0.002**
E velocity (cm/s), mean (SD)	98.2 (29.1)	92.8 (30.9)	0.11
A velocity (cm/s), mean (SD)	77.6 (39.0)	98.1 (36.4)	**<0.001**
Deceleration time (ms), mean (SD)	185.9 (91.4)	246.8 (107.3)	**<0.001**
E/A ratio, mean (SD)	1.60 (0.91)	1.19 (1.20)	**0.002**
E/e’ lateral, mean (SD)	18.3 (11.7)	15.5 (8.3)	**0.006**
TAPSE (mm), mean (SD)	19.2 (4.9)	22.7 (5.1)	**<0.001**

E/A ratio = early-to-atrial filling velocity ratio; E/e’ lateral = early diastolic mitral inflow velocity to lateral mitral annular velocity ratio; GLS = global longitudinal strain; LA = left atrial; LVEF = LV ejection fraction; LV = left ventricular; VTI = velocity-time integral; TAPSE = tricuspid annular plane systolic excursion.

Significant differences (*P* < 0.05) are highlighted in **bold**.

Finally, the RV function assessed by Tricuspid Annular Plane Systolic Excursion was lower in low-LVEF group.

### Changes in LVEF

Following TAVI, 61 patients died during 1 years follow-up with no difference between groups (low LVEF: 7.3% (n = 11) vs normal LVEF: 5.7% (n = 50), *P* = 0.42) (Figure 1), and with 1-year LVEF available in 955 of 968 survivors (Supplemental Figure 1).

**Figure:**
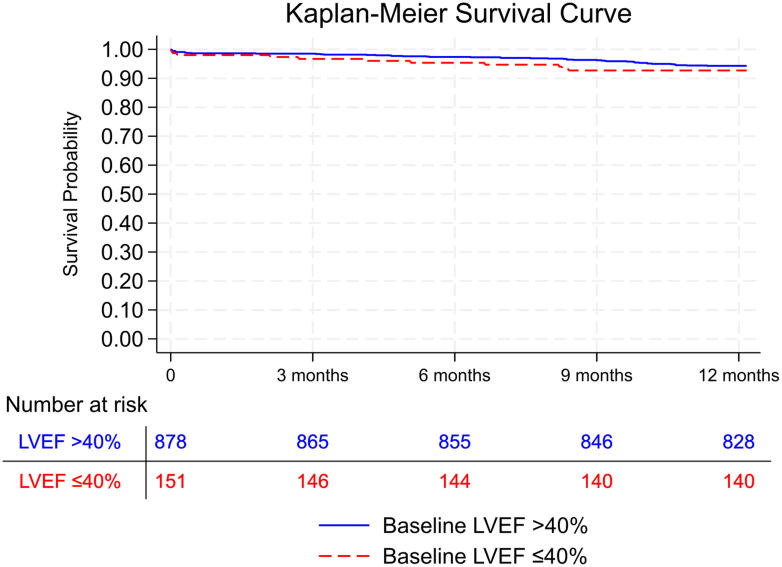
1**Kaplan-Meier Survival Curves for Patients With Low and Normal LV Ejection Fraction** The Kaplan-Meier survival curves depict the 1-year survival probabilities for patients with low LV ejection fraction (≤40%) (red) and normal LV ejection fraction (>40%) (blue). LVEF = LV ejection fraction.

Compared to baseline, LVEF increased to 59.6% ± 12.7% (*P* < 0.001) and 60.5% ± 11.9% (*P* <0.001) after 1 and 12 months after TAVI, respectively. Patients with low baseline LVEF showed significant improvement to 44.8% ± 14.8% 1 month post-TAVI (*P* < 0.001), with further increase to mean LVEF of 52.2% ± 13.6% at 1 year (*P* < 0.001). In contrast, a modest increase was seen in the normal-LVEF group, with LVEF values of 62.2% ± 10.4% and 61.8% ± 11.0% at 1 month and 1 year, respectively (*P* < 0.001) (Central Illustration).

**Central Illustration:**
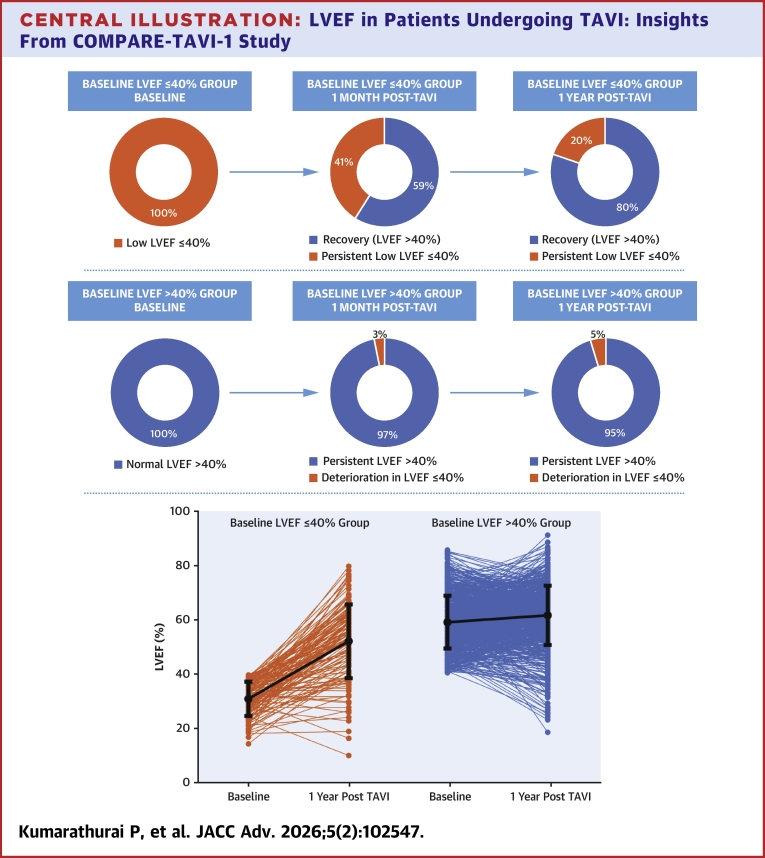
**LVEF in Patients Undergoing TAVI: Insights From COMPARE-TAVI-1 Study** Top: Proportion of patients with persistent LV ejection fraction ≤40 at baseline, 1 month, and 1 year post-transcatheter aortic valve implantation. Middle: Proportion of patients with of deterioration in LV ejection fraction at baseline, 1 month, and 1 year post-transcatheter aortic valve implantation. Bottom: Changes in LV ejection fraction from baseline to 12 months (below) for each patient by LV ejection fraction group. LVEF = LV ejection fraction; TAVI = transcatheter aortic valve implantation.

The increase in LVEF resulted in the majority of patients in the Low LVEF group recovering to LVEF >40% already at 1 month post-TAVI (n = 85, 59%), and at 1 year, this number increased to 110 (80%). Among all cases of improvement, LVEF increased by more than 10 percentage points at 1 year. However, 27 patients (20%) in this group had persistent low LVEF <40% at 1 year following TAVI. In contrast, a small subset of patients with normal baseline LVEF (n = 37, 5%) experienced a deterioration in LVEF to below 40% at 1 year. Among these, the majority (n = 26/37,70%) had a reduction of more than 10 percentage points (Central Illustration).

### Factors associated with persistent low LVEF at 1 year

In a logistic regression analysis a history of CHF, higher NYHA functional class at baseline, lower baseline LVEF and SVi, presence of left bundle branch block, larger baseline LV end-diastolic diameter, lower baseline aortic mean gradient, the presence of pacemaker pre-TAVI, mineralocorticoid receptor antagonists, and sodium glucose co-transporter 2 inhibitor treatment were all associated with persistent low LVEF after 1 year (Table 3). The following variables were associated with persistent low LVEF in a univariate model and remained significant in our fully adjusted multivariate model (in order of largest association first): CHF, pacemaker pre-TAVI, baseline LVEF, LV end-diastolic diameter, aortic mean gradient, and SVi.

**Table 3:** Logistic Regression Analysis. Baseline Covariates Associated With Persistent LVEF≤40% at 1 Year

	Univariate	Model 1	Model 2
Male	1.59 (0.69-3.67)	1.43 (0.58-3.52)	0.86 (0.32-2.32)
Age, per y	0.95 (0.90-1.01)	0.95 (0.90-1.01)	0.96 (0.90-1.02)
CHF	**15.27 (6.07-38.41)**	**19.19 (7.03-52.38)**	**22.60 (7.35-69.51)**
NYHA functional class, per higher class at baseline	**2.16 (1.14-4.09)**	**2.11 (1.10-4.08)**	1.17 (0.61-2.25)
LVEF at baseline	**0.84 (0.80-0.88)**	**0.83 (0.79-0.87)**	**0.84 (0.80-0.90)**
Hypertension	1.10 (0.44-2.77)	1.04 (0.40-2.69)	1.19 (0.44-3.21)
Diabetes mellitus	1.24 (0.52-2.98)	1.06 (0.42-2.68)	0.85 (0.30-2.38)
IHD	1.81 (0.82-4.00)	1.85 (0.81-4.25)	1.14 (0.46-2.78)
Previous AMI	2.45 (0.96-6.21)	2.47 (0.94-6.47)	1.52 (0.55-4.19)
Atrial fibrillation	0.61 (0.28-1.31)	0.66 (0.30-1.47)	0.86 (0.36-2.06)
Pacemaker pre-TAVI	**3.14 (1.29-7.63)**	**3.40 (1.36-8.49)**	**2.86 (1.03-7.95)**
LV end-diastolic diameter, per mm	**1.14 (1.08-1.20)**	**1.16 (1.09-1.23)**	**1.09 (1.02-1.17)**
Mean aortic gradient, per mm Hg	**0.90 (0.87-0.93)**	**0.90 (0.87-0.93)**	**0.93 (0.89-0.96)**
Left bundle branch block	**3.76 (1.54-9.17)**	**4.29 (1.73-10.67)**	1.53 (0.56-4.20)
Right bundle branch block	0.90 (0.21-3.86)	0.91 (0.21-3.96)	0.91 (0.19-4.22)
SVi, per ml/m^2^	**0.91 (0.87-0.95)**	**0.90 (0.86-0.94)**	**0.95 (0.90-0.99)**
ACEI/ARB	1.98 (0.86-4.57)	2.62 (0.96-7.10)	1.71 (0.64-4.55)
Beta-blockers	1.87 (0.86-4.07)	2.05 (0.90-4.67)	1.38 (0.56-3.38)
MRA	**3.86 (1.50-9.90)**	**3.67 (1.37-9.85)**	1.63 (0.53-4.98)
SGLT2i	**3.86 (1.27-11.69)**	**3.54 (1.06-11.88)**	1.16 (0.33-4.14)

This table presents the results of logistic regression analyses examining predictors of persistent low LVEF (≤40%) at 1 year following TAVI. Univariate analysis was performed for each variable, followed by 2 adjusted models: Model 1: age, male sex, weight, hypertension, diabetes. Model 2: model 1 variables + chronic heart failure, NYHA functional class, atrial fibrillation. Results are expressed as ORs with 95% confidence intervals. Statistically significant values (*P* < 0.05) are highlighted.

ACEI = angiotensin-converting enzyme inhibitors; AMI = acute myocardial infarction; ARB = angiotensin receptor blockers; CHF = chronic heart failure; IHD = ischemic heart disease; LV = left ventricular; LVEF = LV ejection fraction; MRA = mineralocorticoid receptor antagonist; RV = right ventricular; SGLT2i = sodium glucose co-transporter 2 inhibitor; SVi = stroke volume index; TAVI = transcatheter aortic valve implantation.

Additional logistic regression models were performed to analyze factors associated with LVEF ≤40% at 1 year regardless of baseline LVEF. In the fully adjusted model, following factors remained independently associated with LVEF ≤40 at 1 year (with largest association first): CHF, left bundle branch block, pacemaker pre-TAVI, diabetes mellitus, LV end-diastolic diameter, baseline LVEF, and mean aortic gradient (Supplemental Table 1).

Excluding patients who died within 30 days after TAVI did not materially change the results, and the same predictors of persistent low LVEF at 12 months remained significant.

### Factors associated with deterioration in LVEF to ≤40% at 1 year

In the fully adjusted model the following factors were associated with deterioration in LVEF to ≤40% at 1 year for patients with baseline LVEF >40% (with largest association first): left bundle branch block, diabetes mellitus, CHF, pacemaker pre-TAVI, LV end-diastolic diameter, and age (Supplemental Table 2).

### Prevalence of IHD across the LV recovery patterns

The prevalence of IHD differed across the 4 LV recovery patterns. IHD was most common among patients with persistent low LVEF (37.0%) and those with deteriorated LVEF at 12 months (40.5%). In contrast, the prevalence of IHD was lower among patients with recovered LVEF (23.6%) and persistent normal LVEF (23.9%).

## Discussion

In this study of 1,031 patients undergoing TAVI, we investigated the prevalence, progression, and prognostic implications of low LVEF ≤40%. Our findings confirm that while the vast majority of patients experienced significant improvements in LVEF over 1 year after TAVI, a subset of patients exhibited persistent LV dysfunction, which is influenced by clinical and procedural factors.

Traditionally LV systolic function has been expressed in terms of LVEF. An LVEF ≤40% is considered significantly reduced,^17^ while LVEF between 40% to 49% and LVEF ≥50% are respectively considered mid-range and normal.^18^ LVEF has a pivotal role in the evaluation of any patient with a cardiac condition, including AS. Despite the almost universal understanding of LVEF, it has important limitations. For decades, it has been well established that LVEF is strongly load dependent^19^^,^^20^ due to the mechanisms described by Starling et al.^21^ Thus, one has to be cautious when interpreting LV function in AS, solely based on LVEF, challenging the prediction of which patients may persist in heart failure after surgery.

Nearly half century ago, Carabello et al^6^ demonstrated among patients with heart failure with severe AS and reduced LVEF that a poor outcome after surgical AV (SAVR) almost was independent of LVEF, and occurred almost solely in those with low transvalvular gradients. We have since demonstrated that LVEF associates with prognosis after SAVR, with an almost linear association between LVEF and outcome.^22^ However, still there is limited data providing information of which patients will remain with systolic heart failure after SAVR or TAVI. It is thus important that we demonstrate that the majority of patients with LVEF <40% prior to TAVI improved in LVEF, suggesting that ventricular unloading after TAVI facilitates reverse remodeling, even in patients with significant pre-existing systolic dysfunction.^23^ Still, despite this improvement, 18% of patients with low baseline LVEF exhibited persistent systolic dysfunction (LVEF ≤40%) after 1 year, raising concerns about the long-term prognosis of this subgroup.

It is therefore important that we demonstrate that in addition to a history of CHF, the presence of pacemaker pre-TAVI, lower LVEF, higher LV end-diastolic diameter, lower mean aortic gradient, and lower SVi are all associated with persistent low LVEF. These findings indicate that while CHF is the primary determinant of LVEF recovery, a combination of structural remodeling and electrical conduction abnormalities also significantly influences postprocedural LV function. Thus, the presence of pacemaker and left bundle branch block at baseline were significant predictor of low LVEF regardless of baseline LVEF, suggests that conduction disturbances or pacing induced ventricular dyssynchrony may limit LVEF recovery. Increased RV pacing rate can lead to impaired ventricular synchronization, resulting in reduced LV filling and in the end LV dysfunction. The electrical and mechanical dyssynchrony induced by RV pacing diminishes stroke volume, contributing to heart failure with reduced ejection fraction. In patients with pacemaker dependency and substantial RV pacing, this dyssynchrony can counteract the benefits of afterload reduction and limit reverse remodeling after TAVI. Our data support previous reports highlighting the adverse impact of pacemaker dependency after TAVI and emphasize the need for careful patient selection and postprocedural monitoring to optimize LVEF recovery.^24^

Similarly, patients with low baseline aortic gradients often exhibit low-flow, low-gradient AS physiology, which is commonly associated with reduced contractile reserve and more advanced structural disease. In these patients, the myocardium may lack the capacity to recover even when valvular obstruction is relieved, resulting in a higher likelihood of persistent LV dysfunction.

Interestingly, diabetes mellitus was independently associated with LV dysfunction regardless of baseline LVEF and with deterioration in LVEF to ≤40% at 1 year, consistent with prior data suggesting that diabetes mellitus promotes adverse LV remodeling.^25^ Additionally, a lower aortic mean gradient was associated with worse LV recovery, reemphasizing the findings from Carabello et al^6^ 5 decades ago.

The observed differences in IHD prevalence across LV recovery trajectories suggest that the underlying myocardial substrate plays an important role in determining the potential for LV functional improvement after TAVI. Patients with persistent low LVEF or deterioration in LVEF exhibited a higher prevalence of IHD. In ischemic cardiomyopathy, myocardial fibrosis and scarring resulting from prior ischemic injury may reduce contractile reserve and limit the myocardium’s capacity to remodel favorably following afterload reduction.

Our findings may have important clinical implications for the management of patients undergoing TAVI: baseline low LVEF should not preclude TAVI referral, as most patients experience substantial recovery over time. Patients with persistent LV dysfunction at 12 months are more likely to have certain pre- and periprocedural risk factors, why recognizing these associations may help guide follow-up and targeted interventions. Prevention of conduction abnormalities post-TAVI is a modifiable factor that may enhance LV recovery, advocating for strategic valve selection and implantation techniques to minimize pacemaker dependency.

### Study Limitations

Several limitations should be acknowledged. The study is observational, limiting causal inferences.

We did not have data on pacing burden, such as the percentage of RV or biventricular pacing, which limits the ability to assess the positive or negative impact of pacing on LV recovery. Furthermore, the number of patients with CRT pacemakers was relatively small, why additional regression analyses were not performed by type of pacemaker.

The definition of IHD was based on documented history of previous acute myocardial infarction, percutaneous coronary intervention, or coronary artery bypass grafting. Thus, patients with stable or medically managed IHD were not classified as having IHD, why there may be an underestimation of the true prevalence of IHD in this cohort.

When retrieving information on medication, the specific clinical indication for treatment was not available. Consequently, drugs commonly used for heart failure management—such as Angiotensin-Converting Enzyme inhibitors or angiotensin receptor blockers, mineralocorticoid receptor antagonists, and beta-blockers—may in some cases have been prescribed for hypertension, rather than for heart failure, and vice versa.

Information on the selection criteria for TAVI candidates with low LVEF—such as the use of dobutamine stress echocardiography, aortic valve calcium score, or other modalities—was not collected, limiting the generalizability of the findings to all patients with low LVEF.

Finally, potential survivor bias must be considered when interpreting our findings. Patients with the most severe LV dysfunction, highest comorbidity, or lowest contractile reserve may be more likely to die before follow-up echocardiography is performed. Thus, these patients are not included in the assessment of LVEF at 1 year. As a consequence, the observed prevalence of persistent LV dysfunction likely represents a conservative estimate, and the true proportion of patients with limited or absent LV recovery after TAVI may be higher. This bias may also partially explain the favorable improvement trajectory observed in the low-LVEF cohort, as the sickest individuals disproportionately contribute to early mortality rather than to follow-up LVEF measures. The relatively small number of patients in the persistent low LVEF group limits the robustness of the logistic regression analyses.

## Conclusions

TAVI is associated with significant improvement in LVEF in patients with reduced baseline LVEF. However, history of CHF, pacemaker pre-TAVI, baseline LVEF, LV end-diastolic diameter, aortic mean gradient, and SVi are associated with lack of recovery in LVEF. One-year mortality rates did not differ between patients with low and normal LVEF at baseline.

Perspectives **COMPETENCY IN MEDICAL KNOWLEDGE:** This study highlights that while TAVI significantly improves LVEF in patients with baseline LVEF ≤ 40%, a subset remains with persistent low LVEF. Identifying predictors such as chronic heart failure, pacemaker implantation, and baseline LVEF may help patient selection and optimize pre- and post-TAVI management strategies.

**TRANSLATIONAL OUTLOOK:** Future research should focus on targeted optimization strategies for patients at risk of persistent LV dysfunction, including TAVI implantation strategies and adjunctive medical therapies to enhance myocardial recovery.

## Funding support and author disclosures

This work was funded by Meril Life Sciences, Vingmed A/S Denmark, the Danish Heart Foundation, and the Central Denmark Region. The funding sources had no influence on the protocol, conduct of the study, or submission of the results for publication. The funding sources had no access to trial data and were not informed about results of the study before submission or presentation of the results. The authors have reported that they have no relationships relevant to the contents of this paper to disclose.
