# Supplementary material for: Left Ventricular Ejection Fraction in Patients Undergoing Transcatheter Aortic Valve Implantation: Insights From the COMPARE-TAVI-1 Study
Source: JACC Adv. 2026 Jan 24;5(2):102547. doi: 10.1016/j.jacadv.2025.102547 (PMC12860949; doi:10.1016/j.jacadv.2025.102547)
Supplement: Supplementary materials [file mmc1.pdf]

Supplemental Table 1. Logistic regression analysis. Baseline covariates associated with low LVEF at 1 Year regardless of baseline LVEF.

**SUPPLEMENTAL TABLE 1**	**Univariate**	**Model 1**	**Model 2**
Male sex	**2.26 (1.25 – 4.09)**	**1.95 (1.04 – 3.67)**	1.43 (0.73 – 2.80)
Age, per year	1.00 (0.96 – 1.04)	1.02 (0.97 – 1.07)	1.03 (0.98 – 1.08)
CHF	**6.42 (3.80 – 10.86)**	**6.55 (3.81 – 11.27)**	**6.14 (3.47 – 10.84)**
**NYHA, per higher class** at baseline	**1.73 (1.14 – 2.60)**	**1.68 (1.10 – 2.56)**	1.18 (0.76 – 1.82)
LVEF at baseline	**0.93 (0.91 – 0.95)**	**0.93 (0.91 – 0.95)**	**0.95 (0.93 – 0.97)**
Hypertension	1.29 (0.69 – 2.41)	1.08 (0.56 – 2.06)	1.19 (0.60 – 2.33)
Diabetes mellitus	**2.25 (1.32 – 3.83)**	**2.16 (1.23 – 3.82)**	**2.09 (1.14 – 3.85)**
IHD	**2.04 (1.21 – 3.45)**	**1.78 (1.03 – 3.06)**	1.44 (0.81 – 2.56)
Previous AMI	**2.53 (1.34 – 4.76)**	**2.14 (1.12 – 4.09)**	1.58 (0.79 – 3.14)
Atrial fibrillation	**0.45 (0.27 – 0.75)**	**0.52 (0.31 – 0.88)**	0.63 (0.36 – 1.09)
Pacemaker pre-TAVI	**2.91 (1.57 – 5.41)**	**2.53 (1.34 – 4.79)**	**2.09 (1.05 – 4.16)**
LV end-diastolic diameter, per mm	**1.14 (1.10 – 1.19)**	**1.16 (1.11 – 1.21)**	**1.12 (1.07 – 1.17)**
Mean aortic gradient, per mmHg	**0.95 (0.93 – 0.97)**	**0.95 (0.93 – 0.97)**	**0.97 (0.95 – 0.99)**
Left bundle branch block	**5.21 (2.88 – 9.40)**	**5.38 (2.93 – 9.88)**	**3.19 (1.65 – 6.16)**
Right bundle branch block	0.54 (0.16 – 1.75)	0.44 (0.13 – 1.44)	0.42 (0.12 – 1.44)
SVi, per ml/m2	**0.94 (0.91 – 0.96)**	**0.94 (0.91 – 0.97)**	0.97 (0.94 – 1.00)
ACEi/ARB	**1.88 (1.09 – 3.23)**	**1.90 (1.02 – 3.55)**	1.47 (0.79 – 2.75)
Betablockers	1.37 (0.83 – 2.28)	1.26 (0.75 – 2.13)	0.89 (0.50 – 1.57)
MRA	**2.93 (1.45 – 5.90)**	**2.72 (1.30 – 5.67)**	1.48 (0.66 – 3.34)
SGLT2i	**4.00 (1.83 – 8.74)**	**2.89 (1.26 – 6.65)**	1.40 (0.58 – 3.41)

Model 1: age, male sex, weight, hypertension, diabetes mellitus

Model 2: model 1 variables + chronic heart failure, NYHA class, atrial fibrillation

Results are expressed as odds ratios (OR) with 95% confidence intervals. Statistically significant values ($p < 0.05$) are highlighted.

Abbreviations; AMI, acute myocardial infarction; ACEi, angiotensin-converting enzyme inhibitors; ARB, angiotensin receptor blockers; ARNi, angiotensin receptor–neprilysin inhibitors ; CHF, chronic heart failure; IHD, Ischemic heart disease; LV, left ventricular; LVEF, left ventricular ejection; MRA, mineralocorticoid receptor antagonist; NYHA, New York Heart Association; RV = right ventricular; SGLT2i, sodium-glucose cotransporter-2 inhibitors; SVi, stroke volume indexed.

Supplemental Table 2. Logistic Regression Analysis. Baseline Covariates associated with deterioration in LVEF to ≤ 40% at 1 Year for patients with baseline LVEF > 40%.

**SUPPLEMENTAL TABLE 2**	**Univariate**	**Model 1**	**Model 2**
Male sex	**2.93 (1.27 – 6.75)**	**2.54 (1.06 – 6.12)**	2.15 (0.87 – 5.30)
Age, per year	1.04 (0.98 – 1.11)	**1.09 (1.02 – 1.17)**	**1.09 (1.02 – 1.17)**
CHF	**3.17 (1.62 – 6.19)**	**2.99 (1.50 – 5.96)**	**2.61 (1.27 – 5.33)**
**NYHA, per higher class** at baseline	1.43 (0.85 – 2.4)	1.40 (0.82 – 2.39)	1.15 (0.66 – 1.98)
LVEF at baseline	0.98 (0.96 – 1.00)	0.98 (0.96 – 1.01)	1.01 (0.98 – 1.04)
Hypertension	1.43 (0.63 – 3.29)	1.11 (0.47 – 2.65)	1.19 (0.50 – 2.86)
Diabetes mellitus	**3.17 (1.63 – 6.17)**	**3.40 (1.66 – 6.96)**	**3.21 (1.54 – 6.68)**
IHD	**2.13 (1.08 – 4.167)**	1.70 (0.85 – 3.40)	1.60 (0.79 – 3.24)
Previous AMI	**2.39 (1.06 – 5.38)**	1.81 (0.79 – 4.17)	1.53 (0.65 – 3.62)
Atrial fibrillation	0.38 (0.19 – 0.73)	0.46 (0.23 – 0.90)	0.52 (0.26 – 1.05)
Pacemaker pre-TAVI	**2.48 (1.10 – 5.58)**	1.82 (0.78 – 4.21)	1.47 (0.62 – 3.47)
LV end-diastolic diameter, per mm	**1.12 (1.07 – 1.17)**	**1.13 (1.07 – 1.19)**	**1.11 (1.05 – 1.18)**
Mean aortic gradient, per mmHg	0.98 (0.96 – 1.01)	0.99 (0.97 – 1.01)	1.00 (0.98 – 1.02)
Left bundle branch block	**5.47 (2.64 – 11.34)**	**5.28 (2.47 – 11.26)**	**4.23 (1.92 – 9.31)**
Right bundle branch block	0.30 (0.04 – 2.24)	0.21 (0.028 – 1.59)	0.21 (0.028 – 1.59)
SVi, per ml/m2	**0.96 (0.93 – 0.99)**	**0.97 (0.94 – 1.00)**	0.99 (0.95 – 1.02)
ACEi/ARB	1.74 (0.86 – 3.51)	1.50 (0.68 – 3.31)	1.28 (0.59 – 2.79)
Betablockers	1.08 (0.56 – 2.08)	0.91 (0.46 – 1.79)	0.70 (0.35 – 1.42)
MRA	2.05 (0.77 – 5.44)	1.99 (0.71 – 5.55)	1.27 (0.43 – 3.75)
SGLT2i	**3.52 (1.30 – 9.53)**	2.38 (0.82 – 6.91)	1.58 (0.52 – 4.79)

Model 1: age, male sex, weight, hypertension, diabetes mellitus

Model 2: model 1 variables + chronic heart failure, NYHA class, atrial fibrillation

Results are expressed as odds ratios (OR) with 95% confidence intervals. Statistically significant values ($p < 0.05$) are highlighted.

Abbreviations; AMI, acute myocardial infarction; ACEi, angiotensin-converting enzyme inhibitors; ARB, angiotensin receptor blockers; ARNi, angiotensin receptor–neprilysin inhibitors ; CRT = cardiac resynchronization therapy; IHD, Ischemic heart disease; LV, left ventricular; LVEF, left ventricular ejection; MRA, mineralocorticoid receptor antagonist; NYHA, New York Heart Association; RV = right ventricular; SGLT2i, sodium-glucose cotransporter-2 inhibitors; SVi, stroke volume indexed.

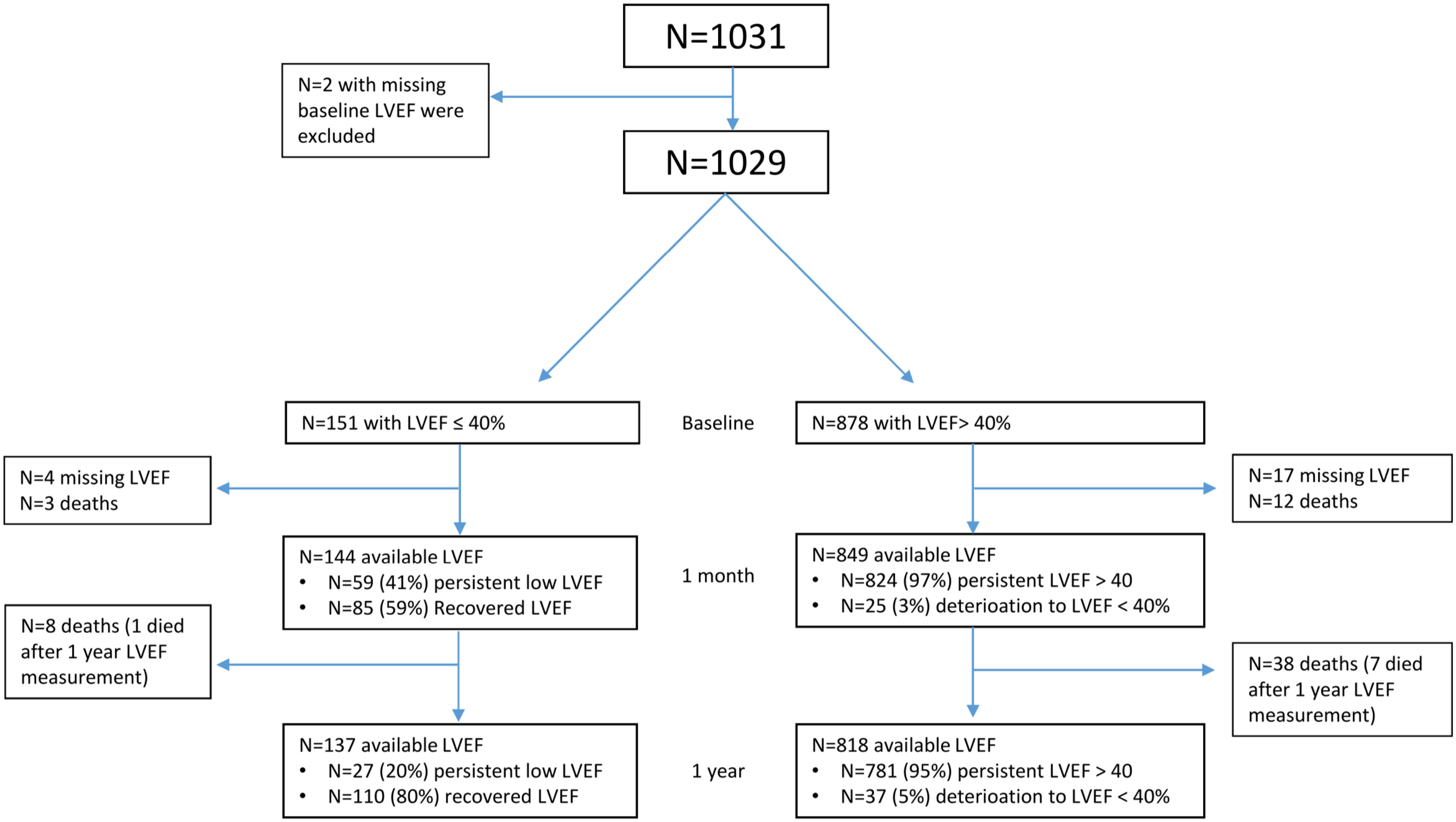
N=1031
N=2 with missing baseline LVEF were excluded
N=1029
N=151 with LVEF ≤ 40%
Baseline
N=878 with LVEF> 40%
N=4 missing LVEF
N=3 deaths
N=17 missing LVEF
N=12 deaths
N=144 available LVEF
• N=59 (41%) persistent low LVEF
• N=85 (59%) Recovered LVEF
1 month
N=849 available LVEF
• N=824 (97%) persistent LVEF > 40
• N=25 (3%) deterioation to LVEF < 40%
N=8 deaths (1 died after 1 year LVEF measurement)
N=38 deaths (7 died after 1 year LVEF measurement)
N=137 available LVEF
• N=27 (20%) persistent low LVEF
• N=110 (80%) recovered LVEF
1 year
N=818 available LVEF
• N=781 (95%) persistent LVEF > 40
• N=37 (5%) deterioation to LVEF < 40%
